# Advancements in Cervical Cancer Screening: Enhancing HPV Testing and Triage Strategies for Improved Risk Assessment

**DOI:** 10.3390/biomedicines13071768

**Published:** 2025-07-18

**Authors:** Yana Merdzhanova-Gargova, Magdalena Ivanova, Angelina Mollova-Kysebekirova, Anna Mihaylova, Nikoleta Parahuleva-Rogacheva, Ekaterina Uchikova, Mariya Koleva-Ivanova

**Affiliations:** 1 Department of Obstetrics and Gynecology, Faculty of Medicine, Medical University of Plovdiv, 4002 Plovdiv, Bulgaria; magdalena.ivanova@mu-plovdiv.bg (M.I.); nikoleta.parahuleva@mu-plovdiv.bg (N.P.-R.); ekaterina.uchikova@mu-plovdiv.bg (E.U.); 2 Department of General and Clinical Pathology, Faculty of Medicine, Medical University of Plovdiv, 4002 Plovdiv, Bulgaria; angelina.mollova@mu-plovdiv.bg (A.M.-K.); mariya.koleva@mu-plovdiv.bg (M.K.-I.); 3 Department of Healthcare Management, Faculty of Public Health, Medical University of Plovdiv, 4002 Plovdiv, Bulgaria; anna.mihaylova@mu-plovdiv.bg

**Keywords:** cervical cancer, HPV, dual-staining test-p16/Ki-67, cytology, liquid-based

## Abstract

**Background/Objectives:** Cervical cancer remains a significant global health issue, with high incidence and mortality rates, particularly in Eastern Europe. Despite the availability of vaccines against human papillomavirus (HPV), regular screening remains crucial for prevention. Testing for HPV, alone or combined with cytology, has become an alternative to traditional methods. However, since many HPV infections are transient, additional tests are needed to identify high-risk cases. **Methods:** This study aims to generate detailed statistical data specific to the Bulgarian population, reinforcing the necessity of incorporating updated European methodologies and algorithms for the prophylaxis and prevention of cervical carcinoma. **Results:** By evaluating epidemiological trends, risk factors, and the effectiveness of current preventive measures, this research seeks to provide a strong foundation for enhancing cervical cancer screening and early detection programs. This method improves triage by identifying women who require further evaluation, ensuring timely referrals for colposcopy or biopsy. **Conclusions:** While liquid-based cytology (LBC) and HPV genotyping improve detection, the introduction of p16/Ki-67 dual staining has enhanced risk stratification, offering higher sensitivity and specificity for detecting high-grade lesions. These advancements are improving cervical cancer screening and patient outcomes.

## 1. Introduction

Cervical cancer is a significant public health issue. In 2012, it was reported as the fourth most common gynecological cancer, with an estimated 528,000 cases and 266,000 deaths globally [1]. In 2018, in Europe, the HPV Information Center estimated that 58,373 women are diagnosed annually with cervical cancer, and 24,404 of those die due to the disease [2].

Bulgaria has a population of 3.06 million women aged 15 and older who are at risk of developing cervical cancer. Current data suggests that each year, 1009 women are diagnosed with cervical cancer, and 503 of them die from the disease. Cervical cancer is the fourth most common cancer among women in Bulgaria and the second most common cancer in patients aged 15 to 44. While data on the HPV burden in Bulgaria’s general population is not yet available, it is estimated that in Eastern Europe, where Bulgaria is located, approximately 9.7% of the female population has cervical HPV-16/18 infections at any given time, with 84.7% of invasive cervical cancers linked to HPVs 16 or 18 [3].

It is now accepted that high-risk human papillomavirus (HPV) is the nearly mandatory viral cause of cervical cancer [4]. This discovery catalyzed the development of two complementary prevention strategies—those that directly target HPV, including the primary prophylaxis with HPV vaccination and HPV testing-based screening for secondary prophylaxis [5].

The reduction in cervical cancer rates is largely attributed to widespread cervical cancer screening programs. Regular screening is the most crucial public health measure for lowering both cervical cancer incidence and related deaths. Tests like conventional cytology (Pap smear), liquid-based cytology, primary HPV testing, and the combined immunohistochemical testing of p16/Ki-67 help detect pre-cancerous changes and the onset of oncogenesis in cervical cells, leading to prevention of invasive cancer or identification of the disease earlier and to more accurate treatment [6,7,8].

According to the data available in the literature, approximately 30% of patients with cervical cancer may have at least 1 previous false-negative cytological result [8]. The conventional Pap test has a false-negative rate ranging from 14% to 33%, with around two-thirds of this being attributed to issues with sampling or slide preparation. Only a small fraction of the sample collected from the patient is placed on the Pap slide, while the rest is discarded with the sampling device. These limitations can result in inaccuracies and unclear diagnoses when using this method. To overcome these challenges, new technologies have been developed, such as the liquid-based smears, despite limited scientific evaluation [9].

Liquid-based cervical cytology was developed to improve the diagnostic reliability of Papanicolaou (Pap) smears. Conventional Pap smears can have false-negative and false-positive results because of inadequate sampling and slide preparation, and errors in laboratory detection and interpretation. However, liquid-based cytology rinses cervical cells in preservatives so that blood and other potentially obscuring material can be separated. It also allows for additional testing of the sample, such as for HPV. The comparative accuracy of each technique has been studied extensively and has yielded conflicting results [10].

The study by Siebers and colleagues helps clarify an important quality issue: that when performed correctly, conventional Pap smears and liquid-based cytology are equivalent for detecting cervical abnormalities. They add that liquid-based cytology often results in fewer unsatisfactory specimens and allows for HPV testing on the same sample. Choice of screening test also depends on the cytology laboratory in which the samples are processed; although liquid-based cytology is more expensive, its ease of use allows laboratories to process slides more quickly and efficiently. For these reasons, liquid-based cytology has virtually replaced conventional Pap smears in the United States. The bigger question, however, is how cytology screening itself will fit into the broader picture of cervical cancer prevention and detection in a rapidly changing environment that includes HPV vaccines and increasingly specific tests for HPV subtypes [11].

Cervical cancer is caused by infection with oncogenic HPV types, of which the International Agency for Research on Cancer (IARC) recognizes 12 HPV types as oncogenic (HPV16, 18, 31, 33, 35, 39, 45, 51, 52, 56, 58, and 59) and 1 HPV type as “probably oncogenic” (HPV68) [12]. It is well established that the HPV type-specific cervical cancer risks vary greatly across HPV types, with the most oncogenic HPV type (HPV16) associated with a >20-fold increased cancer risk and the least oncogenic HPV type (HPV51) associated with a risk increase of only about 1.2-fold [12,13].

The most common histological type of cervical cancer is squamous cell, HPV-associated, of which types 16 and 18 high-risk strains are responsible for about 70% of cervical cancers. The multi-step process of carcinogenesis with the participation of persistent HPV infection is a result of an increase in the expression of the viral oncogenes E6 and E7 in dividing epithelial cells, the so-called transforming infection [14].

The Human PapillomaVirus For Cervical Cancer (HPV FOCAL) Trial included 19,009 women and showed a significant difference in CIN3+ detection at 48 months (2.3/1000 for HPV testing vs. 5.5/1000 for cytology, *p* < 0.01), supporting HPV testing as a more sensitive primary screening tool.

The Human PapillomaVirus FOr CervicAL Cancer (HPV FOCAL) Trial (ISRCTN 79347302) is a large randomized controlled trial, comparing the efficacy of primary HPV testing to liquid-based cytology for cervical cancer screening. It included 19,009 women and concluded that screening with primary HPV testing resulted in significantly lower likelihood of CIN3+ at 48 months compared with cytology (2.3/1000 vs. 5.5/1000) [15].

The elucidation of the role of the virus allows for the introduction of HPV genotyping programs as a future, more sensitive method for stratifying patients and assessing the risk of further oncogenesis [14]. In the current dynamic and evidence-based diagnostic and treatment behavior for HPV-related diseases, numerous recommendations have been developed that encourage the integration of HPV-DNA testing as a primary screening method for cervical cancer prevention [16,17].

The choice of a DNA-based HPV test with high sensitivity is crucial. Tests should be clinically validated and approved for primary cervical cancer screening, as recommended. High HPV sensitivity reduces the risk of missed disease and allows for longer screening intervals [17].

The prospective study—ATHENA, including over 47,000 women, recognizes the importance of detecting high-risk HPV strains. The study found that one in four women positive for the high-risk HPV16 strain will develop cervical pathology within a three-year interval. It also considers the risk of developing precursor epithelial lesions and carcinoma in women with proven HPV 16+ and/or HPV 18+ status, who are cytologically typed as ASC-US or who have had normal cytology, NSIL.

It also found that one in seven women with a normal cytology result from a conventional Pap smear, but with a confirmed high-risk HPV16 strain, has epithelial cervical neoplasia with high malignancy that was missed on cytology [18].

Patients diagnosed with low-grade squamous intraepithelial lesion (L-SIL) or atypical squamous cells of uncertain malignant potential (ASC-US) according to the Bethesda system are recommended for further investigation, including colposcopy and biopsy, to exclude the presence of cervical intraepithelial neoplasia 2 (CIN 2). In young patients, some lesions undergo spontaneous regression, and many HPV strains are transient [8].

However, the specificity of screening women for high-grade CIN (HGCIN) with HPV testing is limited since most HPV infections are transient. Only a low proportion persists and may progress into transforming infections and HGCIN [19,20].

One of the newest approaches to determine the risk of cervical cancer in HPV-positive patients is the dual-staining test-p16/Ki-67 [20]. Combined immunohistochemical testing is used to detect triggered oncogenesis in cervical cells. P16 overexpression is caused by an increase in E7 oncoprotein activity, correlating with persistent HPV infection, while Ki-67 is a marker of cell proliferation. According to recent data, this method has comparable sensitivity, but higher sensitivity compared to HPV testing [8].

The identification of these proteins has proven useful in triaging HPV-positive patients who should be referred for biopsy [19]. A study by Cristina Secosan et al., including 60 women, demonstrated that dual staining alone or in combination with high-risk HPV typing and/or colposcopy had higher specificity than HPV and/or colposcopy [8].

Triage strategies are needed for primary HPV-based cervical cancer screening to identify women requiring colposcopy/biopsy. The impact cohort study by Thomas C Wright Jr et al. assessed the performance of p16/Ki-67 dual-stained immunocytochemistry to triage HPV-positive women and compared it to cytology, with or without HPV16/18 genotyping. The prospective observational screening study involved 35 263 women aged 25 to 65 years. Cervical samples had HPV and cytology testing, with colposcopy/biopsy for women with positive tests. DS showed better risk stratification than cytology-based strategies and provided high reassurance against pre-cancers both at baseline and at 1-year follow-up, irrespective of the HPV genotype. Dual-staining p16/Ki-67 has also been proposed for the triage of patients with ASC-US or L-SIL. DS allows for the safe triage of primary screening HPV-positive women [8,21]. The study found that dual staining was not only a better predictor of lesion development in women with a positive HPV test, but also provided a 5-year free interval if negative, which is more favorable than the 3-year interval of cervical cytology [21].

The PALM study, including 27,349 women, confirmed the higher specificity of p16/Ki-67 dual staining over cytology for the detection of CIN2+ lesions, suggesting a screening method [20,22].

This study aims to generate detailed statistical data specific to the Bulgarian population, reinforcing the necessity of incorporating updated European methodologies and algorithms for the prophylaxis and prevention of cervical carcinoma. By evaluating epidemiological trends, risk factors, and the effectiveness of current preventive measures, this research seeks to provide a strong foundation for enhancing cervical cancer screening and early detection programs. The findings will contribute to the improvement of national healthcare strategies, aligning them with European standards to ensure more effective risk assessment, timely intervention, and better overall health outcomes for women in Bulgaria.

## 2. Materials and Methods

The Preferred Reporting Items for Systematic Reviews and Meta-Analyses (PRISMA) guidelines were followed to perform this work [23].

### 2.1. Literature Search

The authors conducted a thorough and systematic review, gathering all studies on the development of the screening and triage strategies for cervical cancer from databases such as Web of Science, PubMed, Scopus, Google Scholar, and Science Direct. They used the search terms (cervical cancer) or (PAP smear) or (LB cytology) or (HPV testing) or (Dual-staining) and (personalized medicine) to find articles related to the topic. To identify articles focused on screening and triage strategies, they used the search terms (screening) or (triage strategy) and (cervical cancer) or (HPV) or (dual-staining testing). Additionally, they used the terms (cervical cancer) or (HPV) or (p16/Ki67) and (opportunities) or (advantages) and (review) or (systematic review) to locate articles discussing the benefits and advantages of the different strategies.

### 2.2. Eligibility Criteria

The inclusion criteria consisted of articles published from 2010 to 2024, including reviews, systematic reviews, meta-analyses, and full-text articles. Articles were excluded if they were abstracts, short communications, patents and policy reports, case reports, or studies lacking essential information on the screening and triage methods of cervical cancer. We included all articles regardless of language restrictions and compiled a summary of the results.

### 2.3. Exclusion Criteria

Exclusion criteria comprised abstracts, short communications, patents, policy-related documents, case reports, and studies lacking essential information. Articles were included irrespective of language restrictions, and a comprehensive summary of the results was compiled. Duplicate records were removed, and records were excluded due to insufficient data or discrepancies in the study design. During the process, there were other criteria for exclusion: 1. Case reports. 2. Reports of preclinical studies. 3. Reports of complications not directly related to the main objective of the study.

### 2.4. Data Analysis

We utilized Microsoft Office Excel 2010 to create a data extraction form aimed at standardizing the process of data extraction and analysis. The articles were retrieved from various databases and organized in an Excel file, removing any duplicates. Subsequently, the abstracts of the articles were reviewed individually by four authors, who then selected several papers for which the full texts were read independently by all eight authors. This process allowed us to finalize the selection of relevant articles. Few experimental research and prospective studies met all the inclusion and exclusion criteria, as studies on the use of dual-staining p16/Ki-67 are relatively recent. Some sources published before 2010 were also included in this literature review. Once the literature selection was completed, the choices of all authors were reviewed together and discussed until a final consensus was reached.

**Figure 1:**
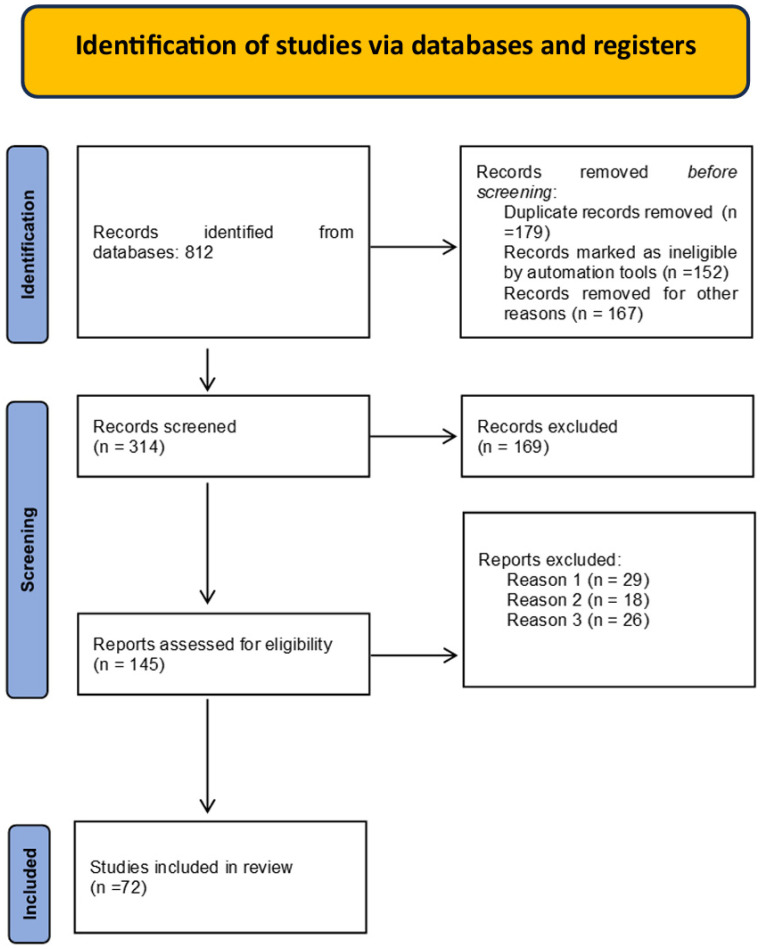
PRISMA flow chart for the selection process of articles.

From the five selected databases, 813 potentially relevant articles were identified. After removing duplicates, a total of 454 studies remained. Following an abstract review, 257 articles were excluded due to insufficient data and/or differing study methodologies. Consequently, 197 full-text articles were analyzed, and 62 were ultimately selected for inclusion. Figure 1 presents the PRISMA flow chart outlining the article selection process for this systematic review.

PRISMA flow diagram. Reasons for exclusion: Reason 1: Systematic review searching/including grey literature. Reason 2: Other types of articles or not a systematic review of empirical studies, for example, articles including reviews, more than 50% descriptive studies or case reports or policy documents. Reason 3: Focus on patient engagement/involvement solely in their individual healthcare decisions. Reason 4: Not explicitly reporting on the involvement of patients and the public in healthcare. Reason 5: No focus on patient/public involvement.

## 3. Results

After carefully examining and analyzing the synthesized scientific literature, we have identified the primary benefits of early endometrial carcinoma detection (Table 1).

To provide a clearer overview of the diagnostic performance of current cervical cancer screening methods, we compiled a comparative summary of their sensitivity, specificity, advantages, and limitations. Table 2 presents this comparison between conventional cytology, liquid-based cytology (LBC), HPV DNA testing, HPV genotyping, and p16/Ki-67 dual-stained cytology.

### 3.1. Screening Accuracy and Diagnostic Value

HPV DNA testing demonstrated the highest sensitivity for CIN2+ detection (90–95%), making it a strong primary screening tool. However, its moderate specificity (85–90%) may lead to false positives, particularly in younger women. Dual-stained cytology (p16/Ki-67) showed both high sensitivity (85–90%) and specificity (90–95%), supporting its role in triaging HPV-positive cases. Conventional cytology, despite high specificity (90–95%), had limited sensitivity (50–60%), while liquid-based cytology offered moderate improvements (65–75%). HPV genotyping enabled precise risk assessment for types 16/18 but lacked coverage of other oncogenic strains.

### 3.2. Age and Personalized Strategies

Screening effectiveness varied by age. HPV testing performed best in women ≥ 30 years due to greater predictive value for persistent infections. Younger women benefited from cytology-based or co-testing strategies to reduce overtreatment. Incorporating molecular markers and genotyping enables personalized screening, tailoring follow-up based on individual risk.

### 3.3. Global Implications and Triage

While conventional cytology remains widely used due to cost and access, newer methods—particularly self-sampled HPV testing and molecular triage—show promise for improving global screening coverage. Dual staining and HPV genotyping enhance risk stratification and reduce unnecessary procedures, but wider adoption requires addressing cost and infrastructure barriers.

## 4. Discussion

### 4.1. Enhanced Screening Accuracy

Regular screening is essential for the prevention of cervical cancer, particularly for early detection and intervention in individuals of reproductive age [24]. Organized screening programs have been shown to significantly reduce cervical cancer mortality by identifying precancerous changes and allowing for timely treatment [25]. The effectiveness of cervical screening varies across different age groups, with tailored approaches providing better outcomes for each demographic. Improved access to cervical cancer prevention and treatment plays a critical role in reducing both morbidity and mortality [26,48]. Screening remains essential not only for early detection but also for monitoring the overall effectiveness of prevention efforts [27,49]. By ensuring accurate and timely screening, we can further reduce the impact of cervical cancer globally [28].

### 4.2. Personalized Medicine Approach

The reduction of both overtreatment and undertreatment is a key benefit of personalized medicine in cervical cancer care [29]. Individualized cotesting strategies allow for more precise screening, enabling targeted therapy tailored to each patient’s specific needs [30]. By calculating personalized risk assessments, healthcare providers can better understand the unique factors that contribute to an individual’s risk of cervical cancer [31]. This approach allows for customized treatment plans based on individual risk factors, improving outcomes and minimizing unnecessary interventions [32,33].

### 4.3. Reduction in Cervical Cancer Cases and Mortality

The importance of ongoing global surveillance of cervical carcinoma is crucial for reducing both incidence and mortality rates [34,35]. Enhanced efforts to predict, prevent, and treat cervical cancer early can significantly improve survival rates. The systematic review provides strong evidence that organized cervical cancer screening effectively lowers mortality rates by detecting precancerous changes and providing timely treatment [36]. Population screening has proven to be an effective strategy, leading to a significant reduction in both morbidity and mortality from cervical cancer [27]. By maintaining surveillance and improving screening access, we can continue to reduce the global burden of cervical cancer [26,37].

### 4.4. Potential for Global Standardization in Screening

Promoting regular screening attendance among women is essential for early identification and better outcomes [28,50]. High detection rates and effective follow-up are critical in lowering cervical cancer mortality. HPV self-sampling for screening increases accessibility, encouraging more women to participate [40,51]. Tracking cervical cancer screening coverage is crucial for assessing progress in the WHO cervical cancer elimination plan, bringing us closer to the worldwide eradication of cervical cancer [39,41,52].

### 4.5. Better Risk Stratification

Despite HPV vaccines, HPV testing will remain central to cervical cancer control. HPV testing, alone or combined with cytology, has become an alternative to traditional screening, but additional tests are needed to identify women with progressing infections or precancer [4,47,53,54]. As vaccination rates rise, screening strategies will need to adapt. Current triage methods for primary HPV screening include genotyping for HPV16 and HPV18 [46,55,56]. Dual staining for p16/Ki-67 provides high performance in risk stratification, offering enhanced sensitivity that significantly improves the detection rates of CIN2+ lesions [42,57]. This method is particularly effective in identifying high-grade cervical lesions, which are crucial for early intervention and prevention of cervical cancer [43]. Furthermore, p16/Ki-67 dual-stained cytology plays a vital role in triaging patients during cervical cancer opportunistic screening, helping to accurately identify those who require further examination or biopsy [44]. This approach enhances the overall accuracy and efficiency of cervical cancer detection and prevention strategies [45].

## 5. Conclusions

Based on the literature review, our team has designed a prospective cohort study involving Bulgarian patients who will undergo liquid-based cytology and HPV testing. High-risk individuals will also receive p16/Ki-67 immunostaining. This study aims to refine national screening protocols by incorporating dual-staining strategies for more accurate risk stratification and early intervention.

Patients who will undergo both liquid-based cytology and HPV status testing simultaneously. Based on the obtained results, high-risk patients will also undergo an additional immunohistochemical testing for Ki-67 and p16. The results of our study will contribute to enriching the knowledge about cervical cancer in the Bulgarian patient population while ensuring a precise and timely therapeutic approach for each individual case.

## Figures and Tables

**Table 1:** Benefits of early endometrial carcinoma detection.

Key Benefits	Significance	Authors
Enhanced screening accuracy	Regular screening is essential for prevention. Early detection and prevention in reproductive age. Organised screening reduces mortality.	Eun T et al. [24] Basoya S et al. [25]
	Effectiveness of cervical screening across different age groups. Improved access to cervical cancer, prevention and treatment. Screening is essential to monitor effectiveness.	Jansen E et al. [26] Sasieni P et al. [27] Bruni L et al. [28]
Personalized medicine approach	Reduction of both overtreatment and undertreatment. Individualized cotesting strategies targeted therapy in cervical cancer calculating personalized risk assessments. Customize treatment based on individual risk factors.	Nygård M et al. [29] Ebadi M et al. [30] Garg P et al. [31] Jones T et al. [32] Quinn M et al. [33]
Reduction in cervical cancer cases and mortality	The importance of ongoing global surveillance of cervical carcinoma. Enhance cervical cancer survival rates. Prediction, prevention, and early treatment for patients. The systematic review provides evidence that organized cervical cancer screening lowers mortality rates. Population screening for cervical cancer led to a significant reduction in both morbidity and mortality.	Singh D et al. [34] Poondla N et al. [35] Hu Z et al. [36] Jansen E et al. [26] Dewar et al. [37]
Potential for global standardization in screening	Encourage women to attend screenings regularly. Good disease-detection rates and good rates of follow-up. HPV self-sampling for cervical cancer screening Cervical cancer screening coverage is key to tracking the WHO elimination plan. Toward the global eradication of cervical cancer.	Landy R et al. [38] Suba E et al. [39] Serrano B et al. [40] Bruni L et al. [28] Canfell K [41]
Better risk stratification	High performance for efficient risk stratification. The enhanced sensitivity of dual staining leads to better detection rates of CIN2+ lesions. Using dual staining for p16/Ki-67 to identify high-grade cervical lesions. p16/Ki-67 dual-stained cytology is employed for triaging in cervical cancer opportunistic screening. Management of HPV-positive women in cervical cancer screening.	Maria Magkana et al. [42] Ouh Y et al. [43] El-Zein M et al. [44] Han Q et al. [45] Wentzensen N et al. [46]

**Table 2:** Comparative data of screening methods for cervical cancer.

Screening Method	Sensitivity for CIN2+ (%)	Specificity (%)	Advantages	Limitations	Key References
**Conventional Cytology**	50–60	90–95	Widely available, low cost	High false-negative rate, limited reproducibility, depends on operator skill	[6,7,9]
**Liquid-Based Cytology (LBC)**	65–75	85–90	Better sample preservation, allows HPV testing on same sample	Higher cost, still limited sensitivity compared to HPV or dual staining	[10,11,47]
**HPV DNA Testing**	90–95	85–90	High sensitivity, allows for longer screening intervals	Low specificity; cannot distinguish transient from persistent infections	[11,17,18]
**HPV Genotyping (16/18)**	~70–80 (for HPV16/18+)	~90	Identifies high-risk infections more precisely	Limited to only two types; does not detect non-16/18 oncogenic strains	[10,11]
**Dual-Staining (p16/Ki-67)**	85–90	90–95	High specificity and sensitivity; effective triage for HPV+ patients	Cost, technical requirements, limited availability in some settings	[8,20,21,42,44]
